# Inflammatory and neuropathological responses to *Vesiculovirus carajas* encephalitis in adult mice: variability, tolerance and resistance

**DOI:** 10.3389/fcimb.2025.1499658

**Published:** 2025-02-26

**Authors:** Maria Sueli Barbosa Cavalcante, Diego Siqueira Santos, Lidineuza Machado Araújo, Priscilla Lieuthier Freitas, Carlos Augusto Moreira Silva, Karina Glazianne Barbosa Carvalho, Marialva Tereza Ferreira Araújo, Eliana Viera Pinto da Silva, Ana Paula Drummond Rodrigues de Farias, Daniel Guerreiro Diniz, Cristovam Wanderley Picanço Diniz, José Antonio Picanço Diniz

**Affiliations:** ^1^ Programa de Pós-Graduação em Neurociências e Biologia Celular, Universidade Federal do Pará, Belém, Pará, Brazil; ^2^ Laboratório de Análises Clínicas do Hospital Universitário João de Barros Barreto, Empresa Brasileira de Serviços Hospitalares, Belém, Pará, Brazil; ^3^ Laboratório de Anatomopatologia, Instituto de Patologia Cirúrgica e Molecular, Belém, Pará, Brazil; ^4^ Laboratório de Microscopia Eletrônica, Instituto Evandro Chagas, Belém, Pará, Brazil; ^5^ Seção de Arbovirologia e Febres Hemorrágicas, Instituto Evandro Chagas, Ananindeua, Pará, Brazil; ^6^ Laboratório de Investigações em Neurodegeneração e Infecção, Hospital Universitário João de Barros Barreto, Instituto de Ciências Biológicas, Universidade Federal do Pará, Belém, Pará, Brazil

**Keywords:** *Vesiculovirus Carajas*, encephalitis, host inflammatory response, cytokines, encephabilitis

## Abstract

*Vesiculovirus carajas* (CARV) is a pathogen with neuroinvasive potential, yet its impact on neuroinflammation and sickness behavior remains poorly understood. In this study, we investigated the neuropathological and immunological responses to CARV encephalitis in adult BALB/c mice. Mice were intranasally inoculated with either infected or uninfected brain homogenates, and clinical, histopathological, and cytokine profiles were analyzed. CARV antigens were primarily detected in necrotic neurons, with prominent microglial activation near the ventricles and blood vessels. By day 10 post-infection, infected mice exhibited significantly elevated levels of MCP-1, IFN-γ, IL-12 p70, TNF-α, IL-6, and IL-10 in the brain, indicating a strong inflammatory response. These findings highlight the inflammatory modulation associated with CARV infection and suggest a hematogenous route of neuroinvasion, distinguishing CARV from other vesiculovirus species. This study provides new insights into the pathogenesis of CARV encephalitis and its potential impact on neuroimmune dynamics.

## 1 Introduction

The *Rhabdoviridae* family, which comprises 434 virus species (International Committee on Taxonomy of Viruses: ICTV, 2024 as of 05/08/2024), is characterized by negative-sense, single-stranded RNA genomes capable of infecting a wide array of hosts, including mammals, birds, reptiles, amphibians, fish, invertebrates, and plants (Kuzmin et al., 2009). In recent years, this viral family has garnered increased epidemiological attention due to associations with fatal encephalitis outbreaks in India (*Vesiculovirus chandipura*) (Ghosh and Basu, 2017; Sapkal et al., 2018) and hemorrhagic fever in Central Africa (Bas-Congo, Ekpoma virus 1 and Ekpoma virus 2) (Kuhn et al., 2020; Stremlau et al., 2015). Additionally, *Rhabdoviridae* members, including the rabies virus (Knobel et al., 2022; Rupprecht et al., 2022) and vesicular stomatitis viruses (Liu et al., 2021), pose significant threats to humans and animals in enzootic regions.

Previous studies have confirmed the natural infection of armadillos (*Dasypus novemcinctus*) by CARV, highlighting the virus’s capacity to infect mammals. The prototype strain of CARV (411391) was first isolated in 1983 in the Serra Norte region of Carajás, Pará, Brazil, during investigations into the circulation of arboviruses in the area. Initially detected in male sandflies, CARV was also identified in pools of female sandflies. Experimental evidence demonstrated transovarian transmission in these vectors, suggesting that this transmission mode may also occur naturally (Travassos Da Rosa et al., 1984; Travassos da Rosa et al., 1992).

Epidemiologically, four Vesiculovirus species New Jersey, Indiana, Cocal, and Alagoas are responsible for vesicular stomatitis outbreaks in cattle, pigs, and horses across the Americas (Pauszek et al., 2008; Rodríguez, 2002). The New Jersey and Indiana serotypes are endemic from southern Mexico through Central and northern South America (Arroyo et al., 2011; Velazquez-Salinas et al., 2018), while Cocal and Alagoas vesiculoviruses have caused outbreaks in Argentina and Brazil (Cargnelutti et al., 2014; Pauszek et al., 2008). Although VSV infections in humans are rare, cases have been reported among farmers and laboratory workers (Reif et al., 1987), including a child in Panama who developed encephalitis following infection with the Indiana vesicular stomatitis virus (Quiroz et al., 1988).

Rhabdoviruses have shown promise as vectors for vaccines and oncolytic therapies, with rabies and vesicular stomatitis viruses being prime examples (Scher and Schnell, 2020; Le Boeuf et al., 2017; Mukesh et al., 2021). The oncolytic potential of this genus, including CARV, is attributed to several factors, such as the relatively small genome, cytoplasmic replication, lack of pre-existing immunity in humans, broad cell tropism, and high viral titers (Hastie and Grdzelishvili, 2012). Comparative genome sequencing of 99 *Rhabdoviridae* members, including CARV, revealed a unique 10,700 nt in the CARV genome, which is distinct from Vesiculovirus Indiana and Vesiculovirus Newjersey sequences (Walker et al., 2015).

Given that RNA virus infections often result in neurological sequelae despite viral clearance, leading to significant motor and cognitive disabilities (Klein et al., 2019; Soung and Klein, 2018), it is crucial to investigate the variability in neuroinflammatory responses and disease progression. This variability may reflect individual differences in tolerance and resistance, with the modulation of the inflammatory response playing a central role (Manglani and McGavern, 2018). Although CARV-induced lethality has been demonstrated in newborn mice, with neuropathological features including lethal encephalitis observed three days post-intranasal infection (Gomes leal et al., 2006), the pathogenic potential of CARV in adult animals remains poorly understood, despite nearly 40 years since its initial isolation. Here, we investigated the neuroinflammatory response and variability in disease progression induced by CARV. Given the limited knowledge of the genetic variability of this virus in nature and its genetic relationship with other Vesiculovirus species, this study aims to utilize CARV as a model of sublethal encephalitis to understand the neuroinflammatory host response better.

## 2 Materials and methods

### 2.1 Ethics statement

This study was reviewed and approved by the Ethics Committee for the Use of Animals (CEUA) of the Evandro Chagas Institute, under certificate CEUA No. 004/2014, issued on April 9, 2014.

### 2.2 Viral strain

Samples of Carajas virus (strain AR 419113) were provided by the Section of Arbovirology and Hemorrhagic Fevers (SAARB) of the IEC.

### 2.3 Viral inoculum

A pilot study was used to determine a suitable infection protocol for generating sublethal encephalitis. The pilot group was used only to define the inoculum associated to clinical signs, the pilot experimental data is described in this section. Three groups of 5 adult BALB/c mice were inoculated intranasally with 0.02 mL of viral suspension at dilutions of 1:2, 1:10 and 1:100. A 1:10 dilution with 2 inoculations separated by a 24-hour interval with observation time windows of 5, 10 and 15 dpi (days post-infection) was used for assessment of the clinical signs and associated neuropathology. Brain suspension from CARV infected mice was obtained from macerate of an infected neonate mouse brain diluted in 1.8 ml of a solution containing phosphate-buffered saline (PBS) pH 7.2, supplemented with 10% bovine albumin (BSA), 100 IU/ml penicillin and 100 mg/ml streptomycin. The macerate was centrifuged for 15 minutes at 15000 g at 4°C, and the supernatant (viral inoculum) was collected and used to induce infection. The titer of the virus used to conduct the study was 3 x 10^9^ PFU.

### 2.4 Experimental groups and timeline

We used 98 female mice aged 56 days. Animals in the infected group had their nostrils instilled with a suspension obtained from infected brain homogenates, and the controls received an equal volume of an uninfected brain homogenate suspension. Pilot assays were performed with 15 animals. To estimate the viral titer, 9 animals were used. Twenty animals were used to study clinical signs (10 control animals and 10 infected animals), and 18 animals were used for the histopathological studies. Another 18 animals were used for immunohistochemistry, and 18 were used for cytokine analysis.

As we intended to cause acute disease induction in 100% of the inoculated animals, we chose to use a 1:10 dilution and two inoculations within 24 hours because in the pilot study, a single intranasal inoculation did not induce infection with evidence of clinical or neuropathological signs. Thus, in the subsequent experiments, two inoculations were adopted to mimic multiple infections, as they may occur in natural conditions.

The control group (CG) and infected group (IG) were observed and euthanized at 5, 10 and 15 dpi. Control animals were inoculated with uninfected brain homogenate. The experimental timeline included the following steps: pilot experiment, pre and post-inoculation titration, study of clinical signs, histology, immunohistochemistry, and cytokine measurements.

### 2.5 Virus titration

Infectious samples were quantified before (viral inoculum) and after inoculation (animals infected at 5, 10 and 15 dpi) by assays in plates (Dulbecco and Vogt, 1953) containing Vero cell monolayers in six-well plates, incubated with serial dilutions (log 10) of 100 µL of the viral sample at 37°C for 1 hour, with gentle agitation every 15 min. After this incubation period, the medium containing non adsorbed virus was replaced by a semisolid culture medium (3% carboxymethylcellulose in medium 199) supplemented with 2% fetal bovine serum, 100 U/mL penicillin and 100 µg/ml streptomycin. After 7 days at 37°C, the cells were fixed and stained with a solution of 0.1% crystal violet, 30% ethanol and 20% formaldehyde in PBS, and the number of cell death zones (plaques) were counted. The viral titer was calculated by multiplying the number of plaques obtained from a given serial viral dilution and the dilution factor, with the result being expressed in plaque-forming units per milliliter (PFU/mL). The detection limit of the assay in this work was 10 PFU/mL.

### 2.6 Clinical signs

Ruffled fur, hunched posture, tremor, reduced exploratory activity, limb paralysis, and circular motion were documented after daily observations were performed at 12-hour intervals for 60 days. The weight of the animals was recorded daily for 20 days.

### 2.7 Perfusion, brain dissection, and cut.

All subjects were euthanized by an anesthetic mixture (ketamine/xylazine (100 mg/kg and 10 mg/kg, intraperitoneally) on the 5th, 10th, and 15th dpi and intracardially perfused with heparinized saline solution (0.9%) followed by aldehyde fixative solution containing 4% formaldehyde and 0.05% glutaraldehyde in 0.1 M phosphate buffer (pH 7.2) for 20 minutes. After perfusion, the brains were dissected from the skull and processed for hematoxylin-eosin staining immunohistochemistry. The perfused brains were sectioned in the horizontal plane (80 µm thick) on a vibratome (Microm HM650V vibrating microtome; Thermo Scientific, Waltham, MA, USA), and anatomical series of sections were collected from all brain regions.

### 2.8 Histology

Brains were washed 3 times for 15 minutes each with 0.1 M phosphate buffer/saline (PBS), pH 7.2, at room temperature, followed by dehydration in increasing dilutions of ethanol (50%, 70%, 90% and 100%) and clearing in a xylene/ethanol solution at a concentration of 1:1, 2:1 and pure xylene. Subsequently, infiltration was carried out by immersion in increasing solutions of paraffin/xylene at a concentration of 1:1 at room temperature. for one and a half hours and two baths of pure paraffin at a temperature of 60°C for two hours each. Horizontal sections (5 µm) were obtained from paraffin blocks using a rotating microtome (Leica RM2235). Sections were mounted on glass slides and stained using hematoxylin–eosin (HE). After cover slipping with embedding medium, sections were observed under an optical microscope (Axiophot - Zeiss), and images were obtained with a digital camera (AxioCam HRC - Zeiss).

### 2.9 Immunohistochemistry

Immunohistochemistry was performed to identify CARV antigens, microglia, and astrocytes. The immunoperoxidase technique was used with the commercial mouse on mouse Vector^®^ M.O.M.™ Immunodetection - M.O.M. (Vector Laboratories), following the manufacturer’s instructions, with some adaptations. The sections were submitted to antigen recovery by incubation in 0.2 M boric acid, pH 9.0, for one hour at a temperature of 65°C to 70°C. Subsequently, the sections were permeabilized with PBS + Triton X-100 (0.5%) in three washes lasting five minutes each. Then, the sections were washed twice with PBS for five minutes.

The sections were then incubated in a solution containing 8% protein concentrate (MOM Kit), 8% horse serum, 3.6% mouse IgG blocking reagent and 0.2% sodium azide in PBS pH 7.4 for the blocking of nonspecific sites for 48 hours. After blocking, the sections were washed three times with PBS containing 8% protein concentrate and 0.2% sodium azide for five minutes each (working solution). This solution was also used for the dilution of the primary antibodies, anti-IBA1 (polyclonal antibody, Wako^©^, # 019-19741), anti-GFAP (monoclonal antibody, Millipore, #MAB360), and anti-CARV (polyclonal anti-CARV were provided by the Evandro Chagas Institute, Arbovirology and Hemorrhagic Fever Section), diluted 1:500, 1:500 and 1:100 respectively. Sections were incubated for 3 days in the primary antibody. After this period, the sections were washed again and incubated for 24 h in secondary antibody (M.O.M. Kit - Biotinylated Anti-Mouse IgG Reagent) diluted in working solution. Subsequently, the sections were washed three times in PBS for five minutes each wash, incubated in hydrogen peroxide (H2O2) diluted in distilled water at a proportion of 1:10 for 15 minutes, and washed again in PBS (three times per five minutes each). Finally, the sections were incubated in ABC reagent (M.O.M. kit) for 3 hours, washed three times in phosphate buffer (0.1 M) for 5 minutes each and incubated in diaminobenzidine (DAB)-nickel developer solution under microscopic observation. The color development was stopped by immersing the sections in phosphate buffer (0.1 M). The sections were coverslipped with Entellan, after which they were analyzed under an Axiophot microscope (Carl Zeiss - Germany) and photographed with an AxioCam HRc digital camera (Carl Zeiss - Germany).

### 2.10 Flow cytometry

Brain homogenates were prepared by maceration at the 5th, 10th and 15th dpi in 0.2 M PHEM buffer (pH 6.9) at a 1:5 dilution, followed by centrifugation for 15 minutes at 15000 g at 4°C. Supernatants were removed and transferred to microtubes. To measure the cytokines IL-12p70, TNF-α, IFN-γ, IL-6, IL-10, and monocyte chemotactic protein (MCP-1), we used the Cytometric Bead Array (CBA) Mouse Inflammation kit (BD Biosciences) following the manufacturer’s instructions. Measurements were performed using a flow cytometer (BD FACSCanto II), and data were obtained using the FACS DIVA software and analyzed using the FCAP Array 3.0 software.

### 2.11 Cytokine measurements (ELISA)

We used the Set Mouse ELISA kit (BD Biosciences) to measure the cytokines IL-1β, IL-12p40, and TGF-β1 in homogenates from control and infected brains, following the manufacturer’s instructions. Ninety-six-well plates were sensitized with capture antibody specific for each cytokine, following dilution curves in the appropriate buffer, and incubated overnight at 4°C. Sensitized plates were washed with a solution containing PBS and 0.05% Tween 20 and then incubated with 200 μL of a blocking solution composed of PBS and 10% fetal bovine serum (FBS) (as recommended by the manufacturer) for one hour at room temperature. The plates were washed again as described above, followed by adding mouse brain suspensions, and incubated for two hours at room temperature.

To quantify the TGF-β1 cytokine, the samples were acidified with hydrochloric acid (HCl) for one hour and then neutralized with 1 N sodium hydroxide (NaOH). After this interval, the plates were washed five times and incubated with biotinylated detection antibody plus peroxidase-linked streptavidin for one hour at room temperature. For the cytokine IL-1β, the detection antibody needs to be added one hour before the streptavidin-peroxidase conjugate (the latter remaining on the plate for 30 minutes along with the samples).

All the plates were washed and incubated with the chromogen tetramethylbenzidine for 15-30 minutes at room temperature under protection from light. After incubation, the reaction was stopped with 2N sulfuric acid (H2SO4) and analyzed in an ELISA reader Model EL 800 - BIO-TEK with a 450 nm filter.

### 2.12 Statistical analysis

Body weight and cytokine production, groups were compared by Mann-Whitney U test, with significance set at p < 0.05. All statistical analyses were performed using GraphPad Prism 10 software.

## 3 Results

### 3.1 Clinical evaluation of infected mice and virus titers in the brain

**Figure 1:**
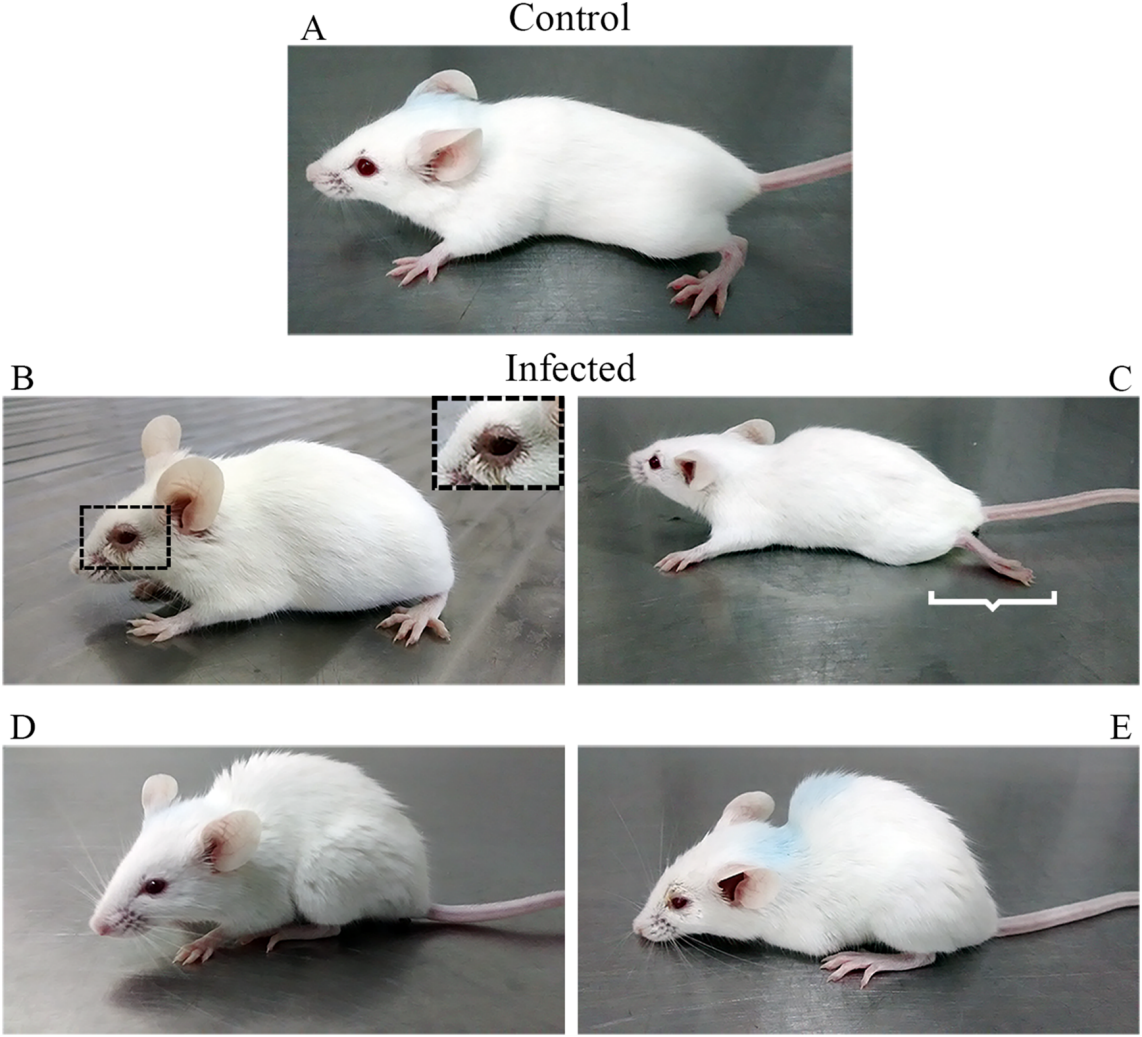
Clinical signs following inoculation of 20 µL in the nostril, twice, 24 h apart, of a suspension of brain homogenate infected with Carajas virus. Control mice **(A)** received an equal volume of uninfected brain homogenate. Conjunctivitis **(B)**, hindlimb paralysis **(C)**, ruffled fur **(D)**, and hunched posture **(E)** occurred from the 10^th^ dpi onward. Hindlimb paralysis was limited to 10% of infected mice.

**Figure 2:**
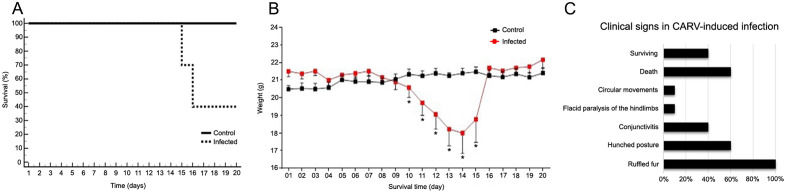
Analysis of the weight, survival loss and clinical signs observed in adult BALB/c mice infected mice with the Carajas virus compared with noninfected mice. **(A)** Analysis of the Kaplan–Meyer survival curve. Log-rank statistical test. p = 0.004. **(B)** Mann-Whitney U test, (*) indicates p value < 0.05 . Body weight and time are indicated on the Y- and X-axes, respectively. **(C)** Clinical signs observed in adult BALB/c mice in CARV-induced infection. 100% of the animals presented hair growth, of which 60% presented progression of clinical signs with death between the 15th and 16th day, 40% presented conjunctivitis, 10% presented circular movements or paralysis of the lower limbs.

Infected subjects showed ruffled fur, conjunctivitis, hunched posture, and flaccid hind limb paralysis (**Figure 1**), followed by death, while the control animals did not show any clinical features. The first clinical signs appeared in all infected mice as ruffled fur between the 9th and 10th dpi, and 60% of these individuals showed progressive aggravation of clinical signs until death between the 15th and 16th dpi (**Figures 2A–C**; **Supplementary Table S1**). Animals surviving CARV infection accounted for 40% of the infected group (**Figure 2A**), all of which showed mild clinical signs that progressively disappeared after 17 dpi. At 20 dpi, these animals were free of clinical signs. The hunched posture was shared by all animals with lethal disease, while the flaccid paralysis of the hindlimbs or circular movements was limited to 10% of the infected mice. Hunched posture, circular stereotyped motions, or total hindlimb flaccid paralysis and death were observed at the 15th and 16th dpi.

The inoculum was 6 × 10^6^ PFU/mL. The post-inoculation viral titer in the brains of animals was 1.7 PFU/mL at the 5th dpi and 1.6 × 10^6^ PFU/mL at the 10th dpi. By the 15th dpi, no plaque formation was observed (0 PFU/mL). Comparative plaque assays at the 5th, 10th, and 15th days post inoculation of different dilutions of a brain homogenate infected with Carajas virus are shown in **Supplementary Figure S1**.

### 3.2 Neuroanatomical distribution of CARV viral antigens

Viral antigen distribution demonstrated by immunohistochemistry showed that the Carajas virus targeted neuronal cells with detailed immunostaining of soma, dendrites, and axons. In general, greater immunolabeling for viral antigens was found in the brain parenchyma of infected animals at 15 dpi. This time point coincides with the most intense histopathological changes and clinical signs. Virus antigens were found close to the vessels and ventricles in the diencephalic (thalamic and hypothalamic regions) and mesencephalic (periaqueductal region and pons) regions. The olfactory bulb and olfactory cortex were the last and less intense CARV immunolabeled regions. As expected, the virus antigens were not detected in any region of the brain parenchyma of the control mice and did not appear in the infected groups during the early stage of the disease (5 dpi- not illustrated). **Supplementary Figures S2** and **Supplementary Figure S3** illustrate CARV immunolabeled neurons in distinct neuroanatomical areas of the infected mice at 10^th^ and 15^th^ dpi respectively. Note that the olfactory bulb is not immunolabeled whereas all other illustrated areas already show CARV immunolabeled neurons. As the disease progresses, immunopathology is aggravated, and this feature is highlighted by intense CARV immunostaining. Note that at 15^th^ dpi, the olfactory bulb remains less stained than all other areas for virus antigens suggesting that neuroinvasion may have occurred mainly by hematogenous pathway.

**Figure 3:**
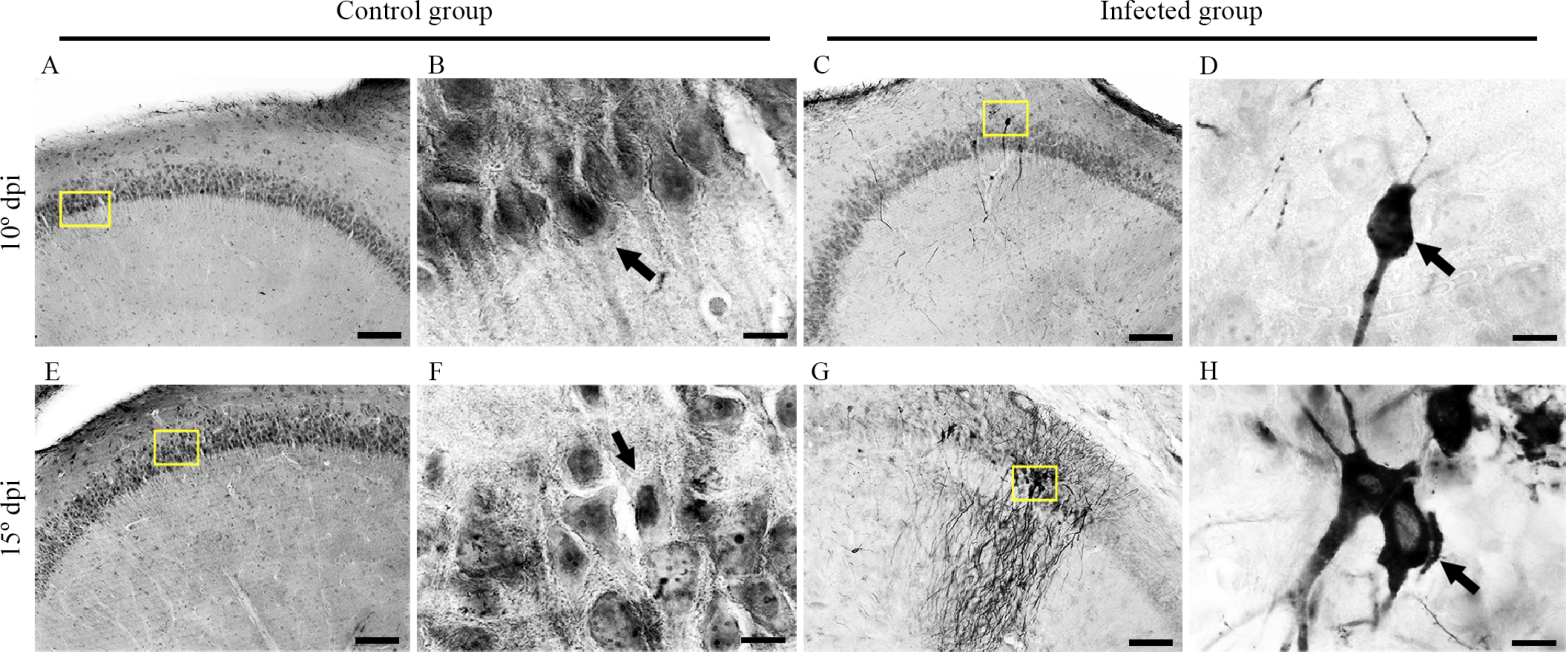
Medium- and high-power photomicrographs from control and infected mice brain sections at 10 and 15 dpi after immunohistochemistry for the detection of viral antigens. Control group sections at 10 dpi **(A, B)** and 15 dpi **(E, F)** showing the absence of labeling in the hippocampus are shown at medium and high power. Infected sections at the 10th **(C, D)** and 15th **(G, H)** dpi show neurons labeled for virus antigens in the hippocampus at medium and high power **(C, G)**, yellow squares. Scale bars: **(A, C, E, G)**, 10 µm; **(B, D, E, H)**, 100 µm.

**Figure 4:**
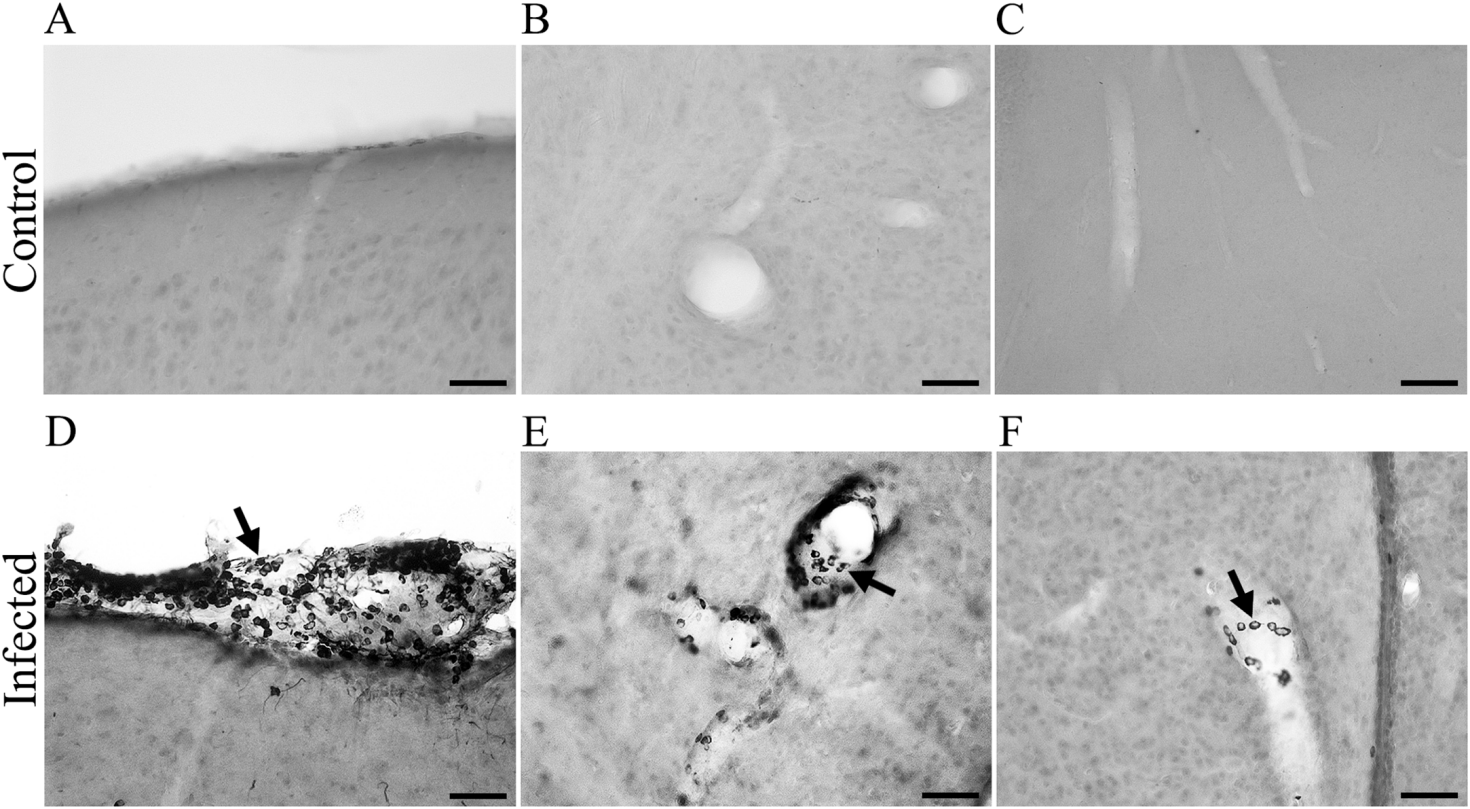
Photomicrographs from brain sections of BALB/c infected mice at 15 dpi, immunolabeled for virus antigens **(A–C)**, showing meningeal leucocyte infiltration in the entorhinal cortex **(A)** and leucocytes in the wall of the mesencephalic and diencephalic blood vessels **(B, C)**. Arrows point to CARV- immunolabeled macrophages inside blood vessels **(D–F)**. Scale bars: **(A–C)**: 25 μm.

**Figure 3** shows higher-magnification photomicrographs of pyramidal neurons of CA2 that were immunolabeled for virus antigens. Meningeal leukocyte infiltration into the entorhinal cortex and leukocyte infiltration into the walls of mesencephalic and diencephalic blood vessels were also labeled for virus antigens at the late stage (15 dpi) (**Figure 4**).

Infected sections at the 10th (C and D) and 15th (G and H dpi) dpi show neurons labeled for virus antigens in the hippocampus at medium and high power (C and G, yellow squares).

### 3.3 Microglial morphological changes

**Figure 5:**
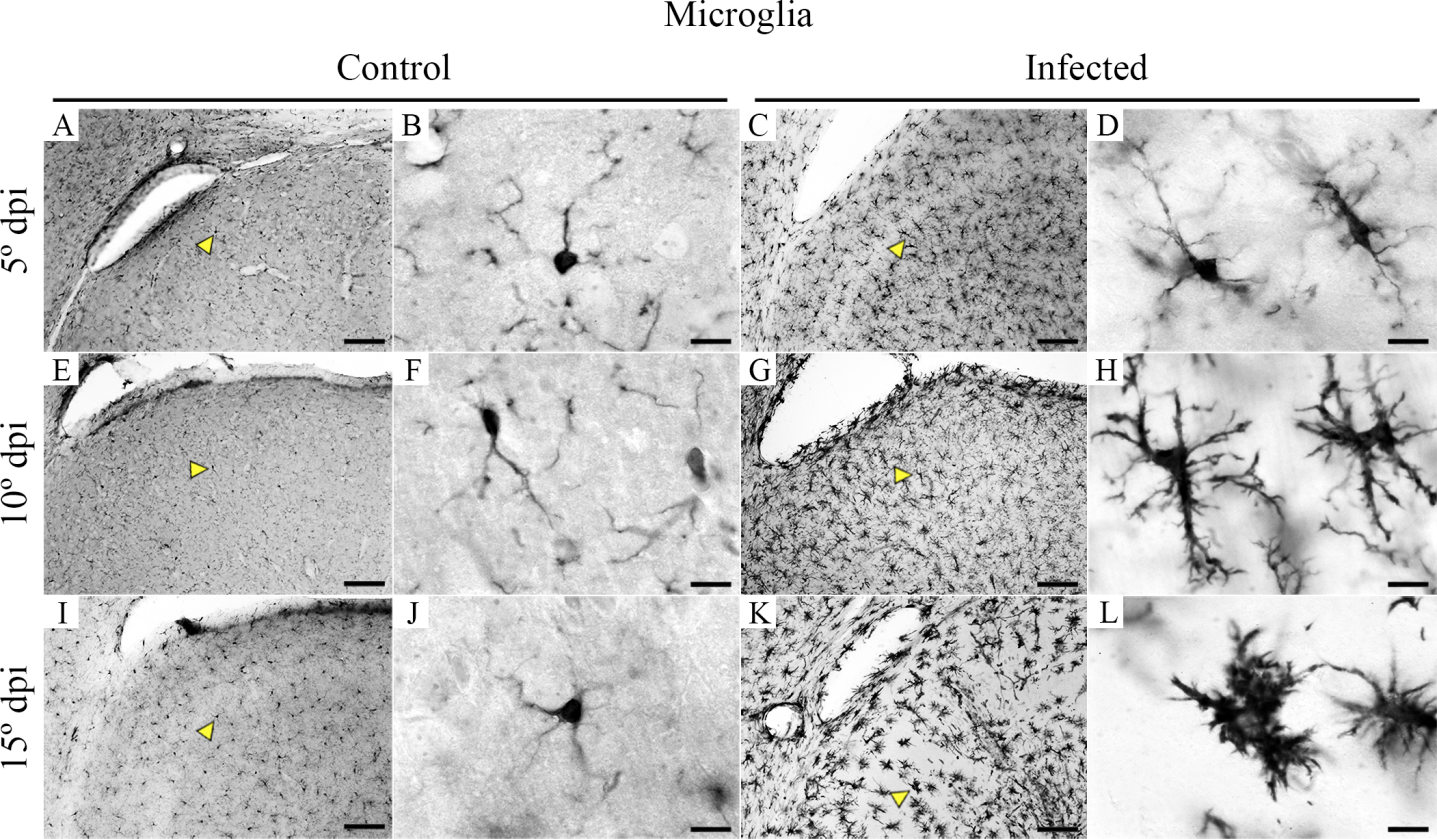
Medium- and high-power photomicrographs of IBA1-microglia, taken from brain sections of control and CARV-infected mouse brains at the 5th, 10th, and 15th dpi to illustrate the morphological changes as the disease progresses. Control IBA1 immunolabeled microglia at 5 dpi, 10 dpi, and 15 dpi are shown at medium and high power in **(A, B**, **E, F, I** , **J)**, whereas progressively activated microglia at the same points in infected mice are shown in **(C,D**, **G, H, K, L)**. Yellow arrowheads indicate medium power, and the microglia are shown at high power. Scale bars: **(A, C, E, G, I, K)**, 10 µm; **(B, D, F, H, J, L)**, 100 µm.

Nonactivated microglia (with long and thinner branches and smaller cell bodies) was observed in all control animals (**Figures 5A, B, E, F, I, J**) while all-time points of infected mice showed progressive microglia activation as disease approach to the later stage where we found thicker and shorter processes near ventricles (**Figures 5C, D, G, H, K, L**).

### 3.4 Histopathological changes

**Figure 6:**
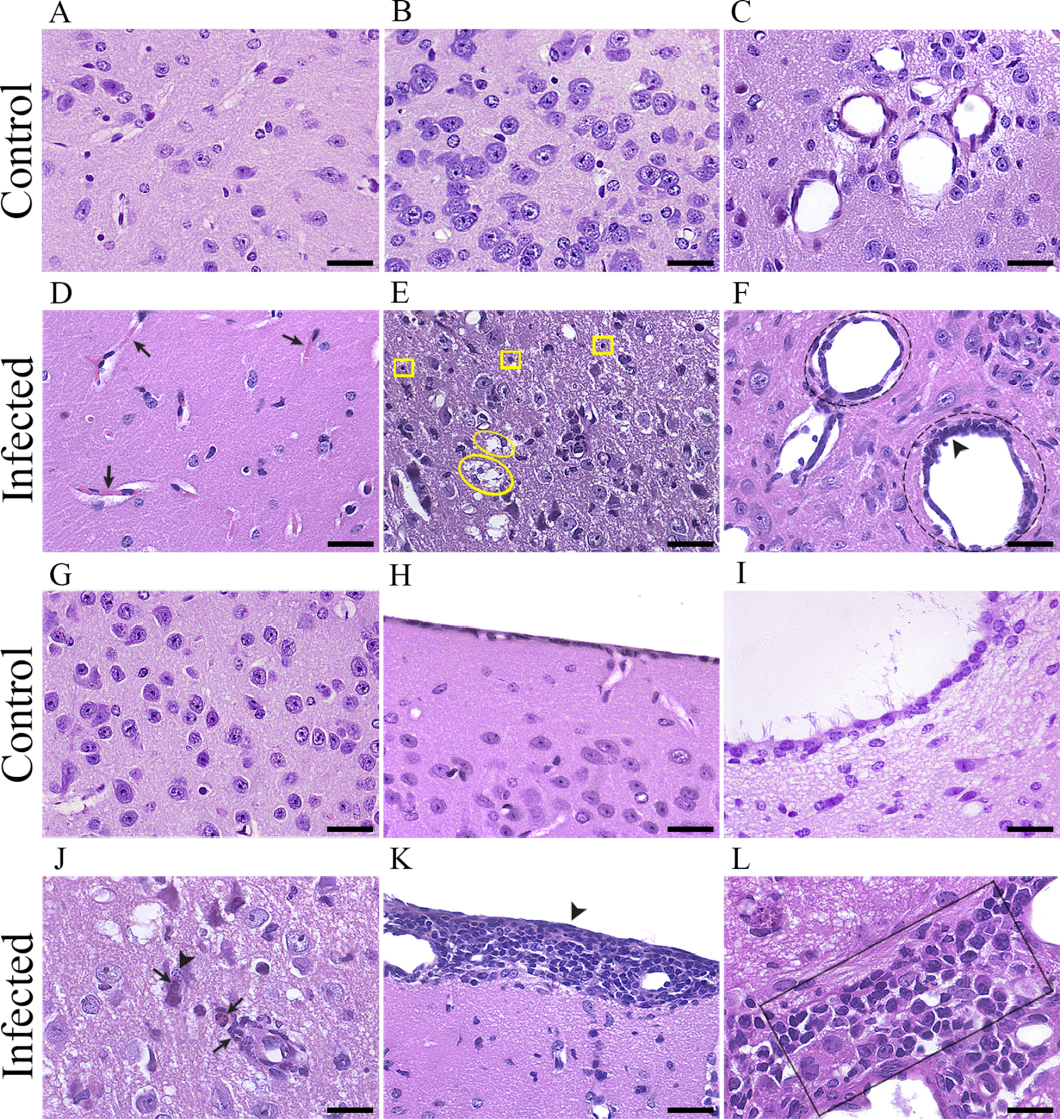
Photomicrographs of histological sections from control **(A-C, G-I)** and infected **(D-F, J-L)** mouse brains stained with hematoxylin/eosin (HE). The control section photomicrograph at 5 dpi illustrates the tissue and vessel integrity in the diencephalon **(A)**. Sections from the infected brain at 5 dpi **(D)** show blood vessel congestion in the diencephalon **(D)**, black arrow). The control section photomicrograph at 15 dpi illustrates the tissue and vessel integrity in the diencephalon **(B, C)**. Sections from the infected brain at 15 dpi **(E-F)**, demonstrating lytic necrosis (**E**, yellow ellipse) and pyknosis **(E)**, yellow squares) in the diencephalon **(E)**. Endothelial proliferation **(F)**, black arrowhead) and vascular ectasia **(F)**, black dotted circle around the vessel at the top). The control section photomicrograph at 15 dpi illustrates tissue and vessel integrity in the diencephalon **(G)**. Sections from the infected brain at 15 dpi **(J)**, demonstrating neutrophilic infiltrate (**J**, black arrows) and karyorrhexis **(J)**, black arrowhead) in the diencephalon. The control section photomicrograph at 15 dpi illustrates the tissue and vessel integrity in the entorhinal cortex **(H)**. Sections from the infected brain at 15 dpi **(K)**, demonstrating meningeal inflammation in the entorhinal cortex **(K)**, black arrowhead). The control section photomicrograph at 15 dpi illustrates tissue and vessel integrity in the 4th ventricle **(I)**. Sections from the infected brain at 15 dpi **(L)**, demonstrating mixed leukocyte infiltrate around the 4th ventricle **(L)**, black rectangle). Scale bars: 30 µm.

Brain sections from control mice were shown to be intact in all regions of the brain parenchyma (**Figures 6A–C, G–I**). At 5 dpi, the tissue and vessel integrity in the diencephalon is illustrated in **Figure 6A**; at 15 dpi, the tissue and vessel integrity in the diencephalon is illustrated in **Figures 6B, C, G**; at 15 dpi, the tissue and vessel integrity in the entorhinal cortex is illustrated in **Figure 6H**; and at 15 dpi, the tissue and vessel integrity in the 4^th^ ventricle is illustrated in **Figure 6I**. Histopathological changes were evident in the infected brain at all three-time points, but they were more intense and frequent on the 15^th^ dpi. At the 5^th^ dpi, congestion of blood vessels in the diencephalon was observed (**Figure 6D**), and at the 10th dpi, mixed leukocyte infiltrate was observed around the ventricles, congestion in the capillaries and pyknosis of the brain parenchyma, mainly around the vessels and ventricles (midbrain). The histopathological changes were intense at 15 dpi (**Figures 6E, F, J–L**), when lytic necrosis and pyknosis were observed in the diencephalon (**Figure 6E**), endothelial proliferation and vascular ectasia near the 4^th^ ventricle (**Figure 6F**), neutrophilic infiltrate in the diencephalon (**Figure 6E**), meningeal inflammation in the entorhinal cortex (**Figure 6K**) and mixed leukocyte infiltration around the 4^th^ ventricle (**Figure 6L**).

### 3.5 Cytokines

**Figure 7:**
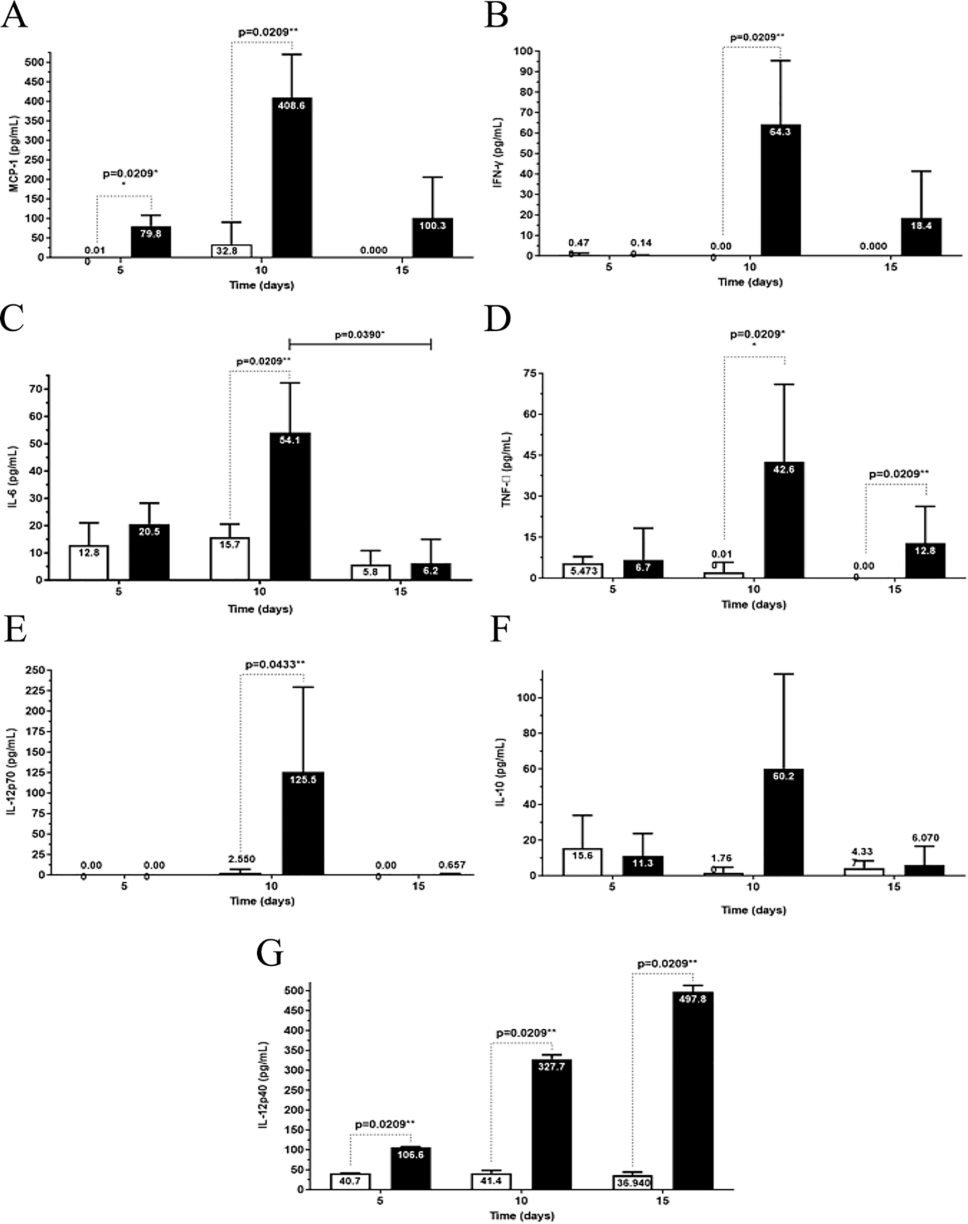
Analysis of cytokine production at 5^th^, 10^th^, and 15^th^ dpi in the brains of albino BALB/c mice infected with Carajas virus. MCP-1 **(A)**, interferon-gama (IFN-γ) **(B)**, IL-6 **(C)**, TNF-α **(D)**, IL-12p70 **(E)**, IL-10 **(F)**, and IL-12p40 **(G)**. The p-value is from the Mann-Whitney U test, * p<0.01 and ** p<0.001.

The chemokine MCP-1 (**Figure 7A**) and the proinflammatory cytokines IFN-γ (**Figure 7B**), IL-6 (**Figure 7C**), TNF-α (**Figure 7D**), and IL-12p70 (**Figure 7E**) in infected mice at 10 dpi were significantly higher than the corresponding cytokine levels in control mice and infected mice at 5 and 15 dpi. At 15 dpi, the levels of inflammatory mediators significantly decreased, returning to baseline values.

The anti-inflammatory cytokine IL-10 (**Figure 7F**, **Supplementary Table S2**) showed no significant differential production of this cytokine in the control and infected groups.

The levels of the cytokine IL-12p40 (**Figure 7G**) showed a significant increase at the three time points post-infection when compared to the levels in the control mice. A progressive increase in IL-12p40 levels was observed during infection (**Figure 7G**).

Regarding the proinflammatory cytokine IL-1β and the anti-inflammatory cytokine TGF-β, there was no significant differential production of these cytokines in the control and infected groups (data not shown).

The greatest MCP-1 chemokine production level was reached at 10 dpi.

## 4 Discussion

In the present study, we explored CARV as a model to investigate the neuroinflammatory response and its modulation in adult BALB/c mice following induced encephalitis. Our findings revealed a complex interplay between the inflammatory response in the brain and the clinical outcomes of CARV infection, particularly in terms of tolerance and resistance to the disease.

As the disease progressed, we observed a significant modulation of the inflammatory response, which was closely associated with the variability in clinical outcomes. Specifically, infected mice exhibited a notable increase in proinflammatory cytokines, including MCP-1, IFN-γ, IL-12p70, TNF-α, and IL-6, particularly at 10 days post-infection (dpi). This peak in cytokine levels coincided with the emergence of severe clinical symptoms, such as sickness behavior and, in some cases, death in the infected group (Maia-Farias et al., 2020; Ghoshal et al., 2007).

The modulation of the inflammatory response appears to be a critical factor in determining the balance between tolerance and resistance to CARV infection. In mice that exhibited a robust proinflammatory response, there was a corresponding increase in microglial activation and leukocyte infiltration within the brain parenchyma. While these immune responses are essential for controlling viral replication, they also contributed to significant neuronal damage and, in some cases, led to the death of the host (Ashraf et al., 2021; Berth et al., 2009; Peng and Wang, 2019). This suggests that while a strong inflammatory response is necessary for resistance to viral spread, it may also predispose the host to increased neuropathology if not adequately regulated (Dantzer, 2001; Dantzer and Kelley, 2007; Kelley et al., 2003). Conversely, some animals displayed a more controlled inflammatory response, characterized by lower cytokine levels at later stages of infection. These mice showed signs of recovery, with a gradual reduction in sickness behavior and eventual resolution of clinical symptoms (Ireland and Reiss, 2004; Komatsu et al., 1999). This pattern is indicative of a more tolerant response, where the immune system effectively manages the infection without causing extensive damage to the host’s neural tissue. This variability underscores the importance of inflammatory modulation as a determinant of disease outcome in CARV-induced encephalitis. The variability in the inflammatory response among different individuals may reflect intrinsic differences in their ability to regulate immune responses, which in turn affects their susceptibility to severe disease. Our findings suggest that animals capable of modulating their inflammatory response effectively are more likely to survive CARV infection, highlighting the potential for therapeutic strategies aimed at fine-tuning the immune response to enhance tolerance and improve clinical outcomes in viral encephalitis (Ahmad et al., 2022; Kucuksezer et al., 2020).

Chauhan et al. (2010) reported that in Vesicular Stomatitis Virus (VSV) infection, viral replication is essential for the robust production of inflammatory mediators in the central nervous system (CNS). These findings align with our observations at 15 days post-infection (dpi), where viral antigens were detected in brain tissue via immunohistochemistry. However, plaque assay results indicated the absence of infectious viral particles (0 PFU) at this stage, suggesting that active viral replication was not occurring. This lack of viral replication may account for the observed selective production of IL-12p40. We acknowledge that our experimental design involved separate cohorts for clinical assessments and cytokine analysis, and we recognize the limitations this imposes on directly correlating cytokine peaks with clinical outcomes. While the initial weight loss around 10 dpi aligns temporally with cytokine elevation, the recorded fatalities, occurring five to six days after cytokine levels peaked, coincide with a return of most cytokines to baseline levels. This distinction underscores the complex interplay between viral activity, immune responses, and disease progression.

The intranasal route of infection is a widely utilized method in studies investigating the effects of Vesiculovirus genus members on the nervous system. Numerous experimental models in the literature have demonstrated the efficacy of intranasal inoculation in inducing lethal infections. Early studies predominantly focused on the Indiana and New Jersey serotypes, with several works supporting the effectiveness of this approach (e.g., Huneycutt et al., 1993; Plakhov et al., 1995; Bi et al., 1995; Cornish et al., 2001). More recent research has extended this method to other vesiculovirus species (e.g., Gomes leal et al., 2006; Maia-Farias et al., 2020; Freitas et al., 2021).

This route was chosen for its relevance in modeling neurotropism and its ability to provide insights into the virus’s impact on the central nervous system (CNS). However, we acknowledge that the natural route of infection for arboviruses may differ and could significantly influence the progression and outcome of the disease. We believe this methodological choice strengthens the relevance of our findings in understanding CNS involvement while also recognizing its limitations in replicating natural transmission pathway.

In conclusion, the modulation of the inflammatory response is a key factor in determining the balance between tolerance and resistance in CARV-induced encephalitis. Our study emphasizes the complexity of the immune response in viral infections and suggests that both the timing and magnitude of cytokine production are critical for determining the course of the disease (Rouse and Sehrawat, 2010; Wu et al., 2014). Future studies should aim to elucidate the molecular mechanisms underlying this balance, which could lead to novel therapeutic approaches for managing viral encephalitis.

## 5 Technical limitations

We utilized plaque assays to quantify viral presence in animal samples, providing robust evidence of disease. However, the lack of a validated real-time quantitative PCR (RT-qPCR) protocol designed explicitly for CARV prevented us from employing this alternative methodology. Although PCR protocols for related vesiculoviruses are available, their sensitivity and specificity for detecting CARV RNA have not been established, which could compromise the accuracy of the results if used. We acknowledge the importance of developing a validated molecular tool for CARV detection and have identified this as a limitation of our study. Furthermore, cytokine levels in the blood were not measured, which imposes additional constraints on our ability to support hypotheses regarding CARV-induced immune responses. This omission highlights the need for further investigations to characterize the systemic immunological effects of CARV better.

## 6 Concluding remarks

In the current study, *Vesiculovirus carajas* served as the investigative tool to examine the neuroinflammatory response associated with encephalitis in adult BALB/c mice. Our observations revealed a progressive manifestation of clinical signs concurrent with an increased dissemination of virus antigens in the brain parenchyma. This escalation was closely linked with notable microglial morphological alterations, leukocyte infiltration, and heightened expression of inflammatory cytokines. Interestingly, microglial activation exhibited greater intensity in proximity to ventricle walls and around blood vessels, suggesting a potential hematogenous route for virus dissemination into the brain. These findings contribute to our understanding of Carajas virus, expanding upon our earlier characterizations of other *Rhabdoviridae* family members such as Maraba, Cocal, and Piry viruses. Given that Carajas virus does not induce human disease and requires only biosafety level 2 containment, its role as a murine model for investigating neuroinflammation and neuronal death holds promise. This model provides an opportunity to explore host-pathogen interactions across molecular, cellular, systemic, and behavioral dimensions.

## Data Availability

The original contributions presented in the study are included in the article/**Supplementary Material.** Further inquiries can be directed to the corresponding author.
