# Supplementary material for: Inflammatory and neuropathological responses to Vesiculovirus carajas encephalitis in adult mice: variability, tolerance and resistance
Source: Front Cell Infect Microbiol. 2025 Feb 26;15:1499658. doi: 10.3389/fcimb.2025.1499658 (PMC11897020; doi:10.3389/fcimb.2025.1499658)
Supplement: Supplementary file 1 [file DataSheet1.docx]

Supplementary Material

**Supplementary Table 1:** Weight (g) in groups Control and Infected according to survival time (days).

| **Time** | **Control** | | **Infected** | |  |
| --- | --- | --- | --- | --- | --- |
| **(Days)** | **n** | **(Weight Mean±sd)** | **n** | **(Weight Mean±sd)** | **p-value** |
| 1 | 10 | (20.524±0.673) | 10 | (21.499±0.946) | 0.3240 |
| 2 | 10 | (20.409±0.862) | 10 | (21.367±0.916) | 0.8533 |
| 3 | 10 | (20.323±0.799) | 10 | (21.507±0.777) | 0.9252 |
| 4 | 10 | (20.443±0.896) | 10 | (20.998±0.900) | 0.9857 |
| 5 | 10 | (20.983±0.803) | 10 | (21.297±0.869) | 0.8017 |
| 6 | 10 | (20.910±0.857) | 10 | (21.387±0.893) | 0.8939 |
| 7 | 10 | (20.801±1.090) | 10 | (21.507±0.877) | 0.5257 |
| 8 | 10 | (20.739±0.793) | 10 | (21.155±0.974) | 0.5491 |
| 9 | 10 | (20.964±0.906) | 10 | (20.901±1.388) | 0.2185 |
| 10 | 10 | (21.208±0.860) | 10 | (20.574±1.738) | *0.0485** |
| 11 | 10 | (21.120±0.828) | 10 | (19.712±2.232) | *0.0082** |
| 12 | 10 | (21.366±0.840) | 10 | (19.052±2.644) | *0.0030** |
| 13 | 10 | (21.238±0.487) | 10 | (18.215±2.974) | *0.0001** |
| 14 | 10 | (21.421±0.819) | 9 | (17.990±3.467) | *0.0006** |
| 15 | 8 | (21.573±0.743) | 7 | (18.770±3.450) | *0.0004** |
| 16 | 5 | (21.406±0.623) | 4 | (21.698±0.597) | 0.9073 |
| 17 | 5 | (21.282±0.582) | 4 | (21.525±0.634) | 0.8123 |
| 18 | 5 | (21.490±0.601) | 4 | (21.705±0.772) | 0.4575 |
| 19 | 5 | (21.282±0.558) | 4 | (21.770±0.737) | 0.4202 |
| 20 | 5 | (21.514±0.605) | 4 | (22.158±1.007) | 0.1510 |

* Mann-Whitney U test


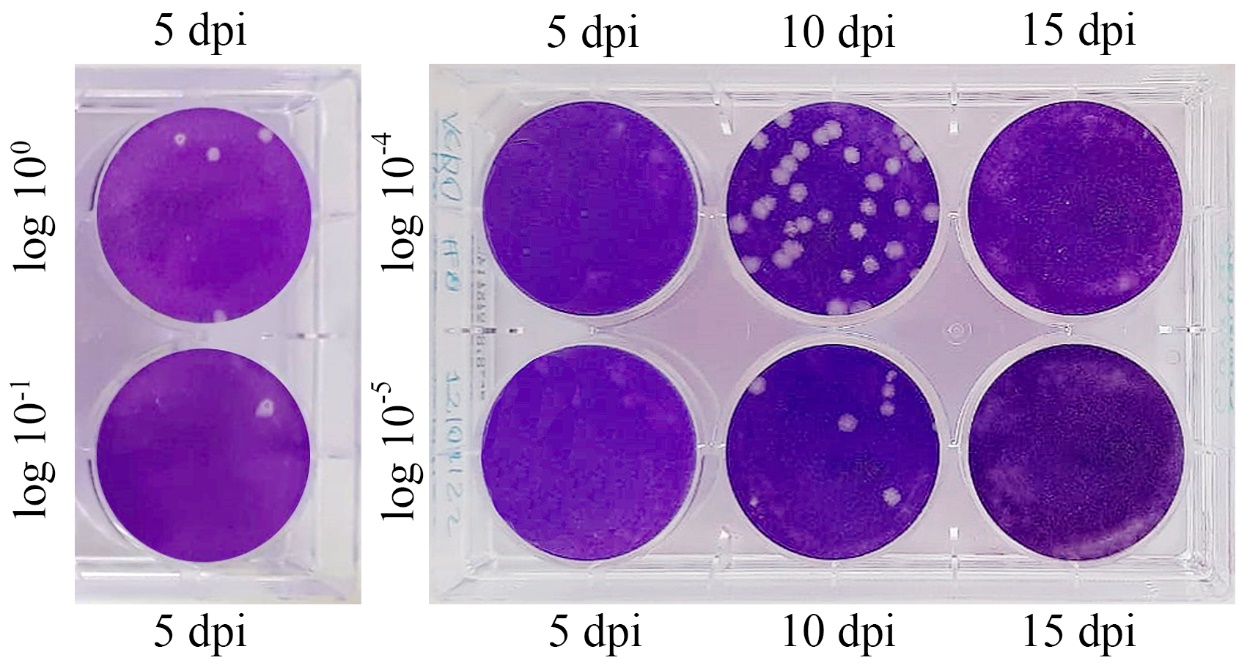


**Supplementary Figure 1.** Comparative pictures of plaque assays at 5th, 10th, and 15th days post-inoculation of different dilutions of an infected brain homogenate with Carajas virus. Plaques were formed until the 10^-5^ dilution at the 10^th^ dpi, whereas at the 5th dpi plaques were formed until the 10^-1^ dilution, and no plaques were formed at the 15th dpi. These findings suggest that the virus load may reached a peak in the brain parenchyma at 10th dpi, and at 15^th^ dpi, the viral load is below the detection limit plaque assay, or alternatively the particles have become non-viable. **Figure S2.** Photomicrographs from infected mouse brain sections at 10 dpi after immunohistochemistry for the detection of viral antigens. Infected sections (**A-C, D-F, G-I, J-L, M-O**) show labeled neurons for virus antigens in the midbrain (**A-C**), diencephalon (D**-F**), fourth ventricle (**G-I**), hippocampus (**J-L**) and olfactory bulb (**M-O**). Macrophages/monocytes are shown in the fourth ventricle (**J-L**). The rectangles indicate the regions of the enlarged photos. Scale bars: (**A, D, J, M**), 350 µm; (B**, E, H, K, N**), 100 µm; (C**, F, I, L, O**), 25 µm.


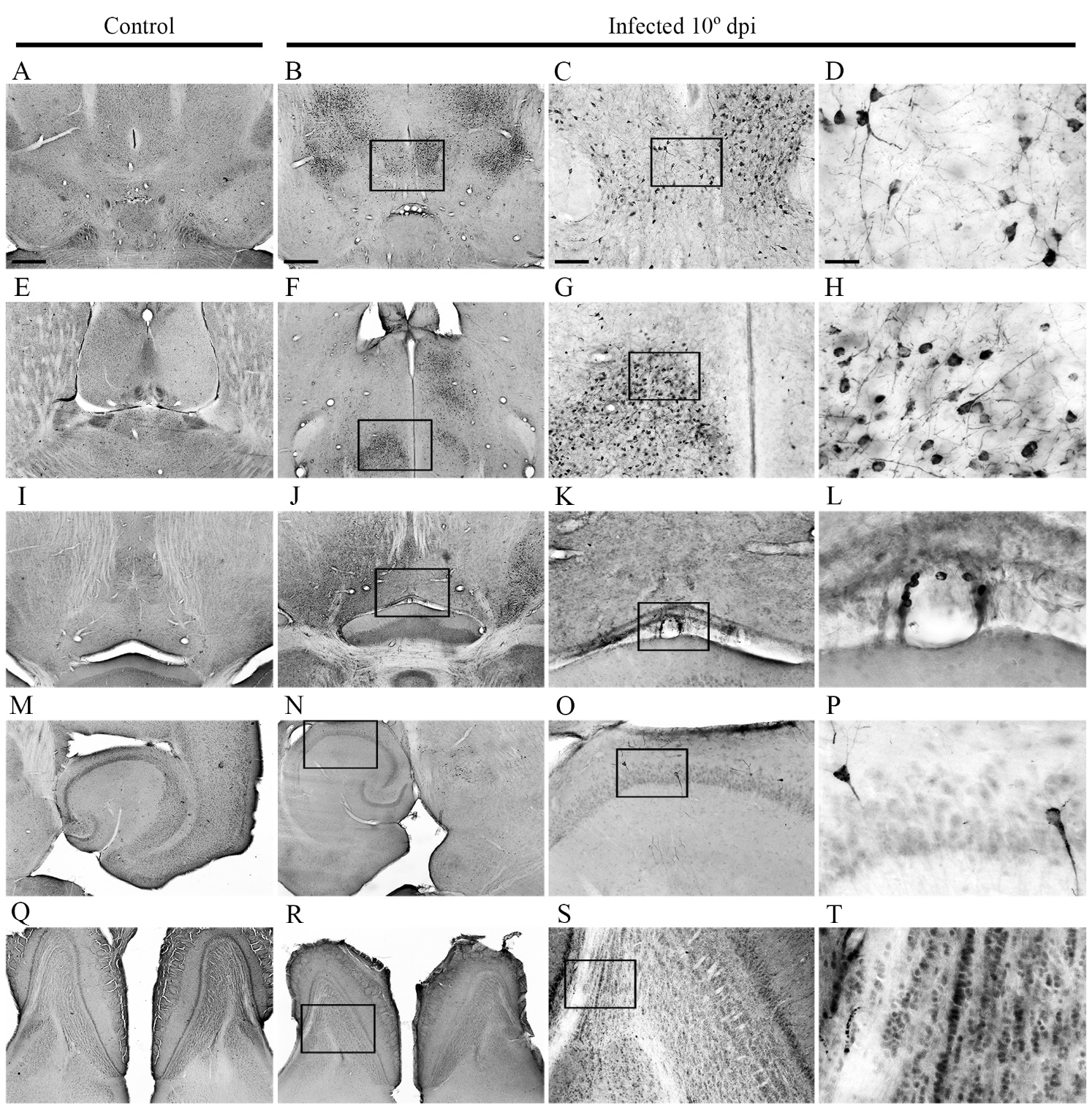


**Supplementary Figure 2.** Photomicrographs from control and infected mouse brain sections at 10 dpi after immunohistochemistry for the detection of viral antigens. Control group sections (**A, E, I, M** and **Q**) show the absence of labeling in the midbrain (**A**), diencephalon (**E**), fourth ventricle (**I**), hippocampus (**M**) and olfactory bulb (**Q**). Infected sections (**B-D, F-H, J-L, N-P, R-T**) show labeled neurons for virus antigens in the midbrain (**B-D**), diencephalon (**F-H**), hippocampus (**N-P**) and olfactory bulb (**R-T**). Macrophages/monocytes are shown in the fourth ventricle (**J-L**). The rectangles indicate the regions of the enlarged photos. Scale bars: (**A, B, E, F, I, J, M, N, Q, R**), 350 µm; (**C, G, K, O, S**), 100 µm; (**D, H, L, P, T**), 25 µm.

**
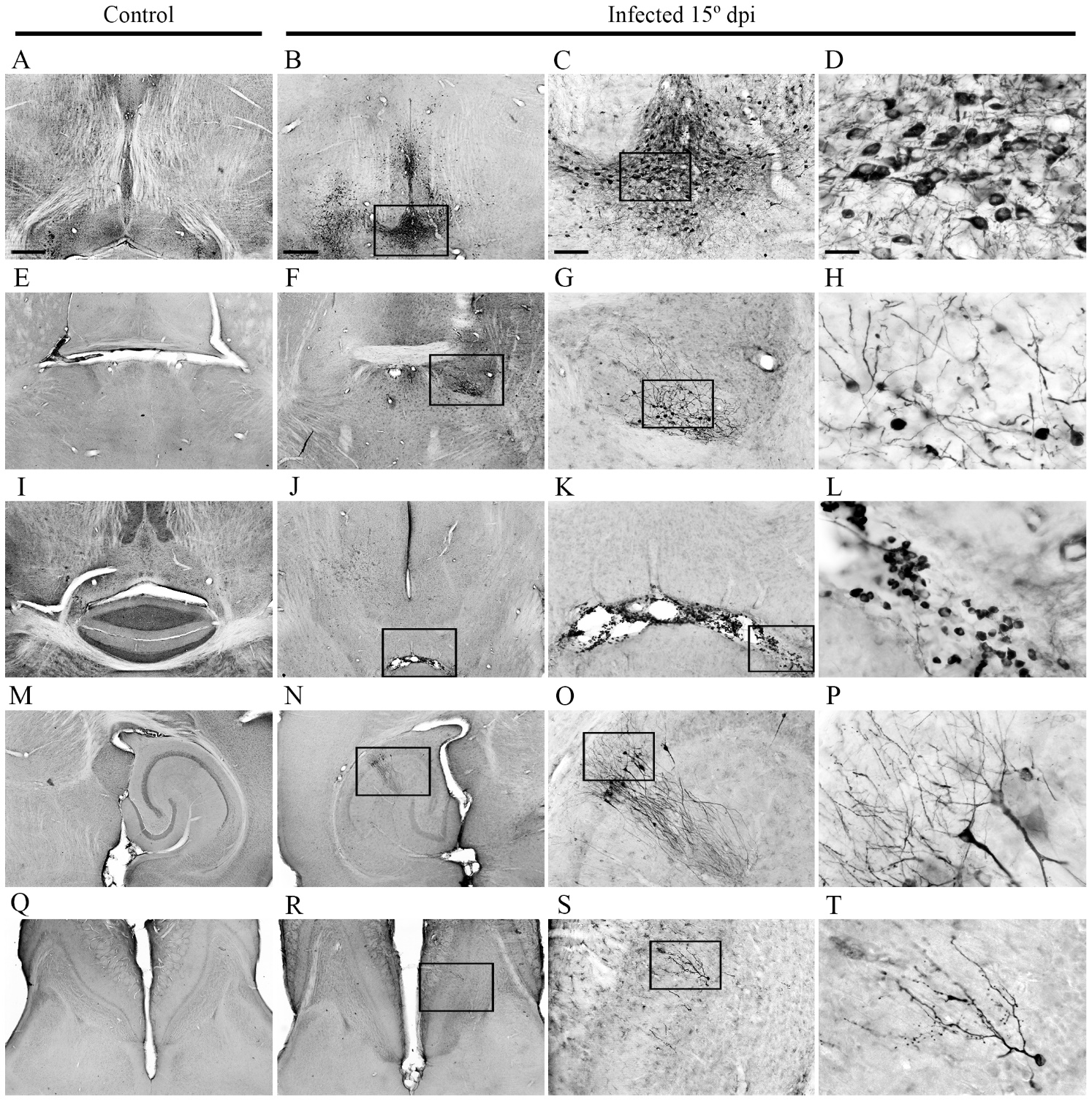
**

**Supplementary Figure 3.** Photomicrographs of brain sections from control and infected mice at 15 dpi after immunohistochemistry for detection of viral antigens. Sections from the control group (**A, E, I, M, Q**) demonstrate the absence of labeling in the midbrain (**A**), diencephalon (**E**), fourth ventricle (**I**), hippocampus (**M**) and olfactory bulb (**Q**) region. Sections from the infected group (**B-D, F-H, J-L, N-P**, **R-T**) show neurons labeled for viral antigens in the midbrain (**B-D**) and diencephalon (**F-H**), hippocampus **(N-P**) and olfactory bulb (**R-T**). Macrophages are shown in the fourth ventricle (**J-L**). The rectangles indicate the regions of the enlarged photos. Scale bars: (**A, B, E, F, I, J, M, N, Q, R**), 350 µm; (**C, G, K, O, S**), 100 µm; (**D, H, L, P, T**), 25 µm.

**Supplementary Table S2**: Cytokines

|  | **Control** | **Infected** | **Control x Infected** |
| --- | --- | --- | --- |
| **Days** | **(Mean±sd)** | **(Mean±sd)** | **p-value** |
| **MCP-1** |  |  |  |
| 5 day | (0.010±0.000) | (79.753±28.069) | 0.0209* |
| 10 day | (32.897±56.979) | (408.627±111.609) | 0.0209* |
| 15 day | (0.000±0.000) | (100.313±105.093) | 0,0833 |
| p-valor Days) | 0,1911 | 0,0665 |  |
| **IFN-γ** |  |  |  |
| 5 day | (0.470±0.814) | (0.140±0.242) | 0,0606 |
| 10 day | (0.000±0.000) | (64.273±31.100) | 0.0209* |
| 15 day | (0.000±0.000) | (18.407±22.957) | 0,2482 |
| p-valor Days) | 0,7408 | 0,0794 |  |
| **IL-6** |  |  |  |
| 5 day | (12.843±8.136) | (20.540±7.635) | 0,1489 |
| 10 day | (15.727±4.764) | (54.083±18.211) | 0.0209* |
| 15 day | (5.793±5.017) | (6.223±8.716) | 0,9999 |
| p-valor Days) | 0,1741 | 0.0390* |  |
| **TNF-α** |  |  |  |
| 5 day | (5.473±2.350) | (6.687±11.582) | 0,2482 |
| 10 day | (2.113±3.660) | (42.557±28.427) | 0.0209* |
| 15 day | (0.000±0.000) | (12.787±13.425) | 0.0209* |
| p-valor Days) | 0,1479 | 0,1479 |  |
| **IL-12P70** |  |  |  |
| 5 day | (0.000±0.000) | (0.000±0.000) | ---- |
| 10 day | (2.550±4.417) | (125.533±103.786) | 0.0433* |
| 15 day | (0.000±0.000) | (0.657±1.137) | 0,5637 |
| p-valor Days) | 0,7408 | 0,0608 |  |
| **IL-10** |  |  |  |
| 5 day | (15.560±18.333) | (11.307±12.432) | 0,5637 |
| 10 day | (1.760±3.048) | (60.157±53.132) | 0,1124 |
| 15 day | (4.337±3.950) | (6.070±10.514) | 0,4705 |
| p-valor Days) | 0,4035 | 0,3932 |  |
| **IL-12p40** |  |  |  |
| 5 day | (40.652±0.643) | (106.561±1.500) | 0.0209* |
| 10 day | (41.410±6.857) | (327.697±11.035) | 0.0209* |
| 15 day | (36.940±6.964) | (497.773±15.428) | 0.0209* |
| p-valor Days) | 0,1801 | 0,1017 |  |

* Mann-Whitney U test**
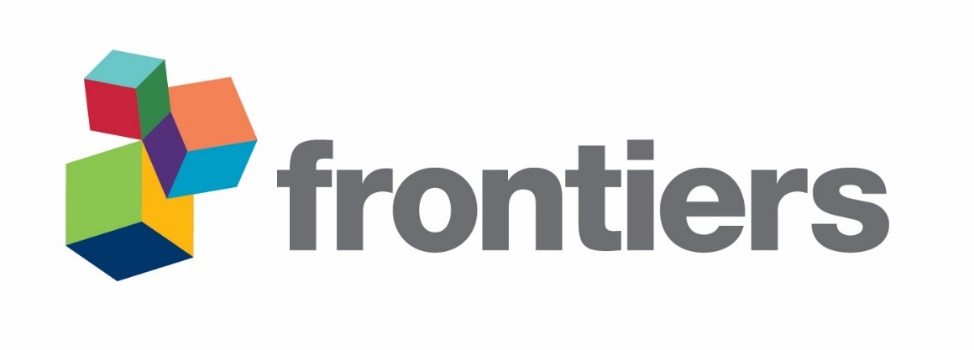
**
